# Haematologic biomarkers and survival in gallbladder cancer: a systematic review and meta-analysis

**DOI:** 10.3332/ecancer.2024.1660

**Published:** 2024-01-30

**Authors:** Rogelio N Velasco, Harold Nathan C Tan, Michael D San Juan

**Affiliations:** 1 Clinical Trial and Research Division, Philippine Heart Center, Quezon City 0850, Philippines; 2 Lung Center of the Philippines, Quezon City 1101, Philippines; 3 Section of Medical Oncology, Makati Medical Center, Makati City 1229, Philippines; 4 Division of Medical Oncology, Philippine General Hospital, Manila 1000, Philippines

**Keywords:** gallbladder neoplasms, inflammation, survival

## Abstract

**Background:**

Gallbladder cancer is a rare malignancy characterised by poor survival with lack of durable response to treatment. Thus, novel biomarkers are needed to prognosticate patients. This systematic review and meta-analysis sought to examine the role of neutrophil-to-lymphocyte ratio, platelet-to-lymphocyte ratio, monocyte-to-lymphocyte ratio, platelet count (PC) and serum immune inflammation index in predicting the survival of patients with gallbladder cancer.

**Materials and methods:**

A systematic search was done using PubMed, Cochrane, ClinicalTrials.gov and Google Scholar for articles published from inception until 8 February 2022. Hazard ratios (HR) with 95% confidence intervals (CI) were pooled and subgroup analyses were conducted according to treatment, region and cut-offs. The primary outcome of interest was overall survival (OS). Data were summarised using RevMan version 5.4.

**Results:**

Twenty studies comprising 5,183 patients were included in the analysis. High neutrophil-lymphocyte ratio (HR 1.72, 95% CI 1.47–2.02), platelet-lymphocyte ratio (HR 1.51, 95% CI 1.33–1.72), monocyte-lymphocyte ratio (HR 1.96, 95% CI 1.46–1.64), PC (HR 1.20, 95% CI 1.02–1.40) and serum inflammation index (HR 1.73, 95% CI 1.36–2.18) were all associated with worse survival. The association was consistent across most subgroups on race and cut-offs with a trend towards poor survival for PC above 252.5.

**Conclusion:**

High neutrophil-lymphocyte ratio, platelet-lymphocyte ratio, monocyte-lymphocyte ratio, PC and SII are associated with worse OS in gallbladder cancer and are potential biomarkers for prognostication. Prospective studies are recommended to further evaluate their use.

## Background

Gallbladder cancer is the sixth most common malignancy of the gastrointestinal tract and the most common biliary tract malignancy accounting for almost 80% of all biliary tract cancers [1]. It is characterised by a dismal survival outcome with poor response to current treatment options such as surgery and systemic treatment [2, 3]. The majority of the patients are diagnosed at an advanced stage or with distant metastasis upon presentation due to the lack of symptoms. The 5-year survival rate for gallbladder cancer patients with distant metastasis is dismal at only 2.7% [4]. Thus, new and effective clinical biomarkers are needed to predict outcomes to optimise treatment outcomes.

Inflammation is a known hallmark of cancer development and progression. Within the tumour microenvironment, cytokines, chemokines and other molecules from both malignant and host cells facilitate invasion, angiogenesis and spread. Systemic inflammation likewise involves cytokines, inflammatory proteins and immune cells. Both the local and systemic inflammation involve cross signalling and plays a crucial role in cancer biology. Agents have thus been developed targeting the inflammatory process such as anti-angiogenic agents such as bevacizumab, anti-CTL4A antibodies such as ipilimumab and antiinterleukins [5].

Tumour-derived proteins have the capacity to increase myelopoieses. This increase myelopoieses can result in tumour angiogenesis, invasion and distant spread. Neutrophils and its precursors–myelocytes and promyelocytes–may also be increased in cancer-related bone marrow dysfunction. Monocytes which reside in tissue have been shown to be associated with increased tumour stage among colon cancer patients. The neutrophil-to-lymphocyte ratio (NLR) has been utilised as a negative prognostic marker in various types of malignancies such as prostate cancer and colon cancer [7, 8]. The platelet-to-lymphocyte (PLR) ratio has also been utilised in prognosticating malignancies such as non-small cell lung cancer [9]. The monocyte-lymphocyte-ratio (MLR) has been explored as a marker of poor prognosis on cervical and colorectal cancer [10, 11].

Platelets produce platelet-derived endothelial cell growth factor, which may contribute to the induction of mitosis and angiogenesis [14]. An elevated platelet count (PC) has also been shown to be a poor prognostic factor among patients with pancreatic cancer, colorectal cancer and gallbladder cancer [12–14]. The systemic immune-inflammation index (SII), an index of platelets, neutrophils and lymphocytes, has been reported to be prognostic in several cancers such as prostate cancer, colorectal cancer and pancreatic cancer [15–17].

NLR, PLR and MLR as prognosticators in biliary tract cancer have been explored in various studies, albeit with conflicting results [4, 18–20]. The prognostic utility of PC and SII likewise have not been explored in a meta-analysis. Due to the conflicting results among different studies and the absence of a consensus on their prognostic role, we performed a systematic review and meta-analysis to examine the predictive role of NLR, PLR, MLR, PC and SII on the overall survival (OS) of patients with gallbladder cancer. These indices can be used in predicting high-risk gallbladder cancer for which more aggressive treatment and monitoring may be considered.

## Methods

### Search strategies

**Figure 1.**
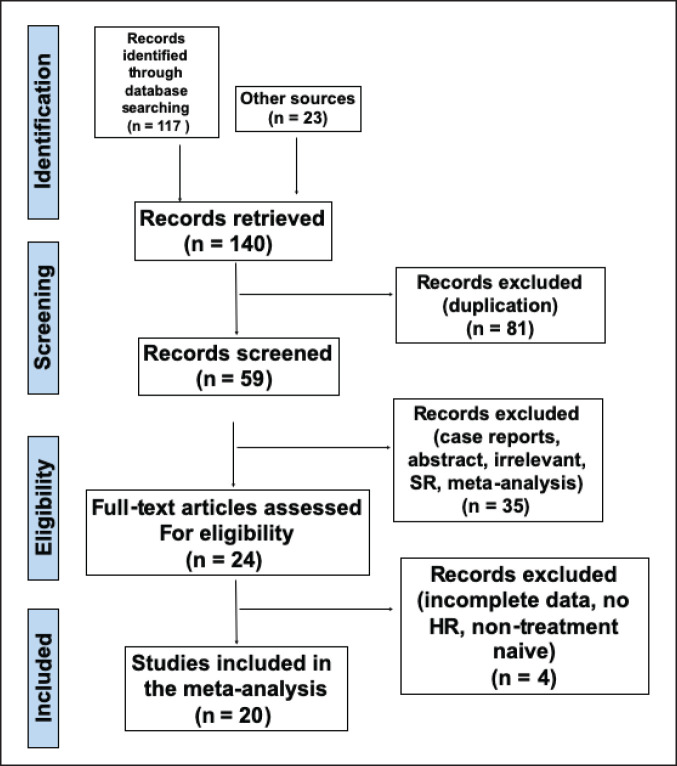
PRISMA flow diagram of the included studies in the meta-analysis.

A systematic literature search was independently conducted by two investigators (Harold Tan and Rogelio Velasco) using the PubMed, Web of Science, Google Scholar and Cochrane Library databases from inception until 8 February 2022, to obtain relevant articles. Studies were retrieved using the following search terms: (‘neutrophil-to-lymphocyte ratio’ OR ‘neutrophil-lymphocyte ratio’ OR ‘NLR’ OR ‘platelet-to-lymphocyte ratio’ OR ‘platelet-lymphocyte ratio’ OR ‘PLR’ OR ‘monocyte-to-lymphocyte ratio’ OR ‘monocyte-lymphocyte ratio’ OR ‘MLR’ OR ‘platelet count’ OR ‘PC’ OR ‘systemic immune inflammation index’ OR ‘SII’) AND (‘gallbladder cancer’ OR ‘gallbladder carcinoma’). The references of each candidate article were also searched to identify other studies that can be included in the analysis. The full search strategy is shown in Figure 1.

### Selection criteria

Two independent authors screened the possible articles for inclusion if they met the following criteria: (1) full-text journal articles written in English involving human subjects with histopathologically confirmed gallbladder cancer; (2) articles with data on NLR, PLR, LMR, MLR, PC or SII with corresponding cut-off values; (3) studies with reported associations between the haematologic biomarkers and prognosis expressed as hazard ratios (HR) and 95% confidence intervals (95% CI) as measures of association. Articles were excluded if they fulfilled any of the following: (1) studies with incomplete data to calculate HRs and 95% CI; and (2) case reports, review articles, conference abstracts, expert opinions and commentaries. For articles with multiple publications, only the latest and most comprehensive publication was considered. Furthermore, authors of articles with incomplete data were contacted by the investigators.

### Data extraction and quality assessment

The risk of bias for each study was assessed using the Newcastle-Ottawa Quality Assessment Scale for Cohort Studies (NOS) [21] by two independent authors (Rogelio Velasco and Harold Tan) and all disagreements were settled in consensus with a third independent author (Michael San Juan). Briefly, the NOS includes eight items, classified into three domains: selection of study participants, comparability of cohorts and ascertainment of outcome. Scores were defined as high quality (>7), moderate quality [5–7] or low quality (<5). The following were obtained from each study: first author, geographic region, year of publication, total number of patients, study design, tumour stage, treatment given, cut-off used, follow-up data and the outcome of Cox regression analysis using HRs and 95% CI obtained from univariate or multivariate analysis, the latter of which was preferred. NLR, PLR and MLR values were defined as the ratio of the absolute neutrophil count and the absolute lymphocyte count (NLR), the ratio of the absolute PC and the absolute lymphocyte count (PLR), and the ratio of the absolute monocyte count and the absolute lymphocyte count (MLR) in the peripheral blood. SII was defined as the absolute PC multiplied by the NLR. The primary outcome assessed was OS, characterised as the time from histopathologic diagnosis of gallbladder cancer to death from any cause.

### Statistical analysis

HR and 95% CI were extracted from the included studies and combined using the generic inverse variance method using Review Manager 5.4. A HR of more than one indicated worse OS above the biomarker cut-off, while a HR of less than one denoted improved survival below the biomarker cut-off. Heterogeneity was determined using the Higgins *Ι*^2^ statistic and Cochran’s *Q*. A fixed-effects model was used to determine pooled HR when *I*^2^ is less than 50% or *p* is more than 0.10. Otherwise, we employed the random-effects model [22]. When *I*^2^ was more than or equal to 50%, subgroup analyses were analysed to determine possible sources of heterogeneity. Subgroup analyses were performed according to the median cut-off used and geographical region (Asia versus other regions). For analyses that included ten or more studies, publication bias was evaluated using a funnel plot.

The present systematic review and meta-analysis were conducted in accordance with the Preferred Reporting Items for Systematic Reviews and Meta-Analysis (PRISMA) guidelines and the Cochrane Handbook for Systematic Reviews of Interventions.

## Results

A total of 140 articles were gathered from electronic databases based on the specified search strategy. Upon removal of articles not related to gallbladder cancer, a total of 24 articles were obtained. Records unrelated to prognostication and those with incomplete data were likewise excluded. Full-text articles were reviewed based on the aforementioned inclusion and exclusion criteria. A total of 20 articles were then included in the final analysis (Figure 1).

The included studies comprised 5,183 patients, with sample sizes ranging from 93 to 691 (Table 1). Studies were published from 2014 to 2021 and were conducted predominantly in China (*n* = 14) with the rest of the studies from Korea (*n* = 2), and the USA (*n* = 4). Treatment received varied between studies with diverse cut-offs, and treatment rendered (i.e., surgery only, chemotherapy only or a combination approach). Based on the Newcastle-Ottawa Scale for Cohort Studies, the majority of the studies were of low to moderate quality (Table 1).

### Haematologic biomarkers and OS

#### Neutrophil-lymphocyte ratio

**Supplementary Figure 1.**
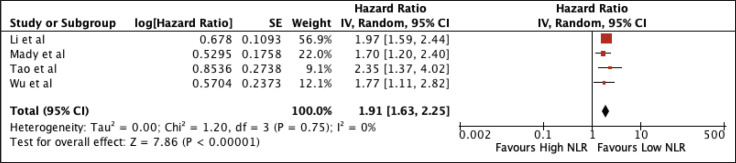
Sensitivity analysis for NLR excluding studies with poor quality.

**Figure 2.**
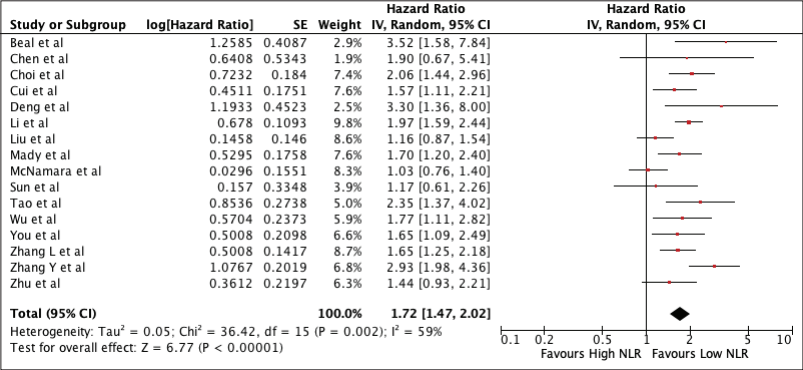
Forest plot of studies exploring the relationship between NLR and OS.

Sixteen studies were included encompassing 3,806 patients were included in the analysis. Figure 2 shows the association of elevated NLR with worse OS (HR 1.72, 95% CI 1.47–2.02, *p* < 0.00001) with high heterogeneity (*I*^2^ = 59%). Subgroup analyses performed according to the geographic region still showed worse OS on both the Asian (HR 1.77, 95% CI 1.51–2.06) and non-Asian subset (HR 1.65, 95% CI 0.95–2.89). Using the median cut-off value of 2.675, there was worse OS on both subgroups (NLR ≤ 2.675: HR 1.965, 95% CI 1.66, 2.32; NLR > 2.675: HR 1.53, 95% CI 1.23, 1.89). The heterogeneity was lower on the cut-off value less than the median, indicating cut-offs as a cause of heterogeneity. A sensitivity analysis excluding all studies with poor quality still confirmed its prognostic use (Supplementary Table 1 and Supplementary Figure 1).

**Figure 3.**
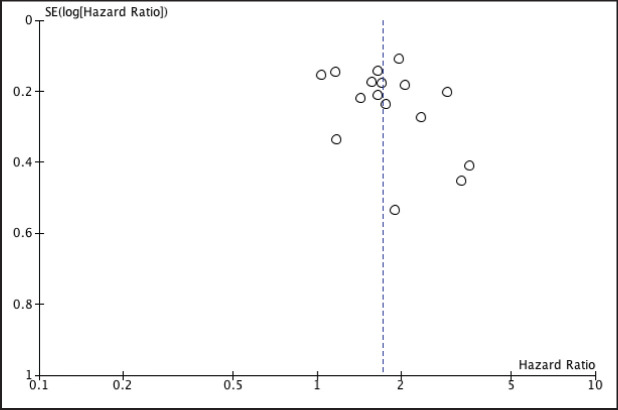
Funnel plot investigating publication bias in studies involving NLR.

To account for publication bias, a funnel plot was constructed showing no evidence for publication bias in the relationship between NLR and OS (Figure 3).

#### Platelet-lymphocyte ratio

**Supplementary Figure 2.**
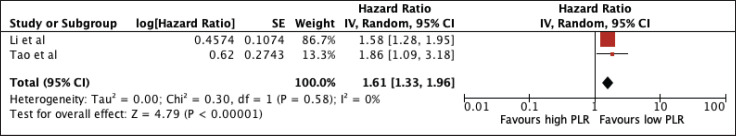
Sensitivity analysis for PLR excluding studies with poor quality.

**Figure 4.**
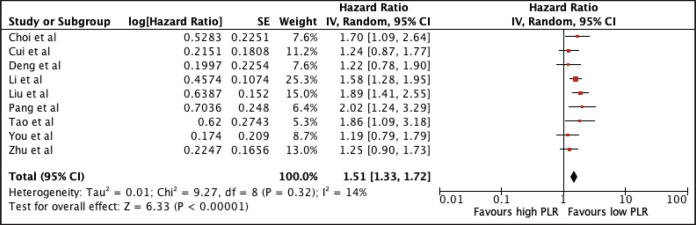
Forest plot of studies exploring the relationship between PLR and OS.

Nine studies with a total of 2,171 patients were included in the analysis. Elevated PLR was associated with poor OS (HR 1.51, 95% CI 1.33–1.72, *p* < 0.00001) (Figure 4). A subgroup analysis using the median cut-off value of 140.305 still showed poorer OS with a higher PLR (PLR ≤ 140.305: HR 1.87, 95% CI 1.52, 2.29; PLR > 140.305: 1.37, 95% CI 1.19, 1.58). Notably, there was no heterogeneity among studies using the cut-off below and above the median. A sensitivity analysis excluding all studies with poor quality still confirmed its prognostic use (Supplementary Table 1 and Supplementary Figure 2).

#### Monocyte-lymphocyte ratio

**Supplementary Figure 3.**
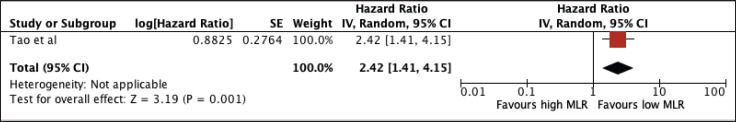
Sensitivity analysis for MLR excluding studies with poor quality.

**Figure 5.**
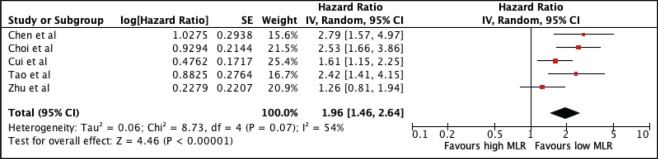
Forest plot of studies exploring the relationship between MLR and OS.

769 patients from five studies were included in the analysis of MLR and OS. A high NLR was associated with poor OS (HR 1.96, 95% CI 1.46–2.64, *p* < 0.00001) with significant heterogeneity (*I*^2^ = 54%) (Figure 5). The prognostic utility of MLR was consistent among studies using the median cut-off of 0.29 (MLR ≤ 0.29: 1.96, 95% CI 1.23, 3.11; MLR > 0.29: 2.01, 95% CI 1.18, 3.42). Subgroup analysis was not possible since all studies are Asian. A sensitivity analysis excluding all studies with poor quality still confirmed its prognostic use (Supplementary Table 1 and Supplementary Figure 3).

#### Platelet count

**Supplementary Figure 4.**
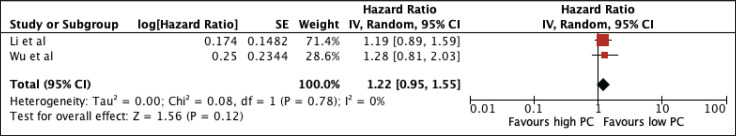
Sensitivity analysis for PC excluding studies with poor quality.

**Figure 6.**
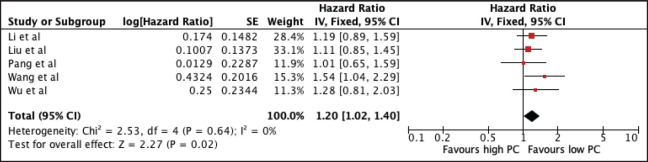
Forest plot of studies exploring the relationship between PC and OS.

1,618 patients from five studies were included in the analysis of MLR and OS. Figure 6 shows the association of elevated NLR with worse OS (HR 1.20, 95% CI 1.02–1.40, *p* = 0.02) with no heterogeneity. The prognostic significance of increased PC and poorer OS was consistent among studies utilising a cut-off below the median 252.5 × 10^9^ (PC ≤ 252.5 × 10^9^: 1.24, 95% CI 1.01, 1.51) and a trend towards poor survival among studies with cut-offs >252.5 × 10^9^ (HR 1.13, 95% CI 0.89, 1.45). A sensitivity analysis excluding all studies with poor quality still confirmed its prognostic use (Supplementary Table 1 and Supplementary Figure 4).

#### Serum immune-inflammation index

**Supplementary Figure 5.**
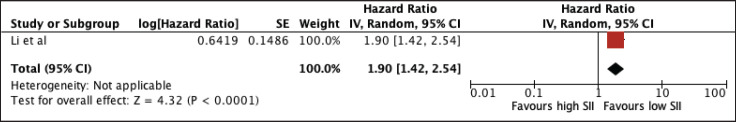
Sensitivity analysis for SII excluding studies with poor quality.

**Figure 7.**
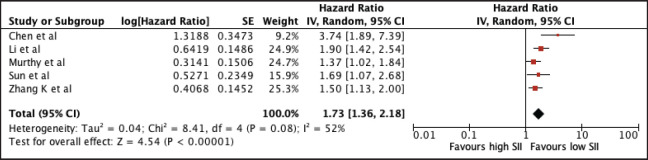
Forest plot of studies exploring the relationship between SII and OS.

1,764 patients from four studies were included in the analysis of MLR and OS. The pooled analysis showed the association between elevated NLR with worse OS (HR 1.73, 95% CI 1.36–2.18, *p* < 0.00001) (Figure 7). A subgroup analysis among Asian studies showed consistent prognostic use of SII and a trend towards poor survival among non-Asian studies (Asian: 1.77, 95% CI 1.51, 2.06; non-Asian: 1.65, 95% CI 0.95, 2.89). Using the median cut-off value of 555, there was poor survival with increased SII regardless of the cut-off value (SII ≤ 555: 1.58, 95% CI 1.33, 1.86; SII > 555: 2.41, 95% CI 1.12, 5.22). A sensitivity analysis excluding all studies with poor quality still confirmed its prognostic use (Supplementary Table 1 and Supplementary Figure 5).

## Discussion

This systematic review meta-analysis investigated the prognostic significance of haematologic indices in gallbladder cancer. Our results show that among the 20 studies included in the analysis, NLR, PLR, MLR, PC and SII are all associated with poor OS and can potentially be used as prognostic indices in gallbladder cancer. To our knowledge, this is the first meta-analysis evaluating the prognostic significance of pretreatment PC and SII on gallbladder cancer. In addition, we updated the meta-analysis by Xu *et al* [38] on NLR, PLR and MLR.

The process of inflammation elicits both pro- and anti-inflammatory responses through the release of mediators. Neutrophils, key sources of cytokines, are associated with tumour progression [39, 40]. Platelets have also been shown to be potent sources of cytokines that can bind various growth factors such as vascular endothelial growth factor and fibroblast growth, both of which are key players in tumour angiogenesis, proliferation and metastasis [41–43]. Monocytes have been shown to secrete various pro-inflammatory cytokines which have been shown to adversely affect prognosis in cancer [12, 44]. Lymphocytes, most notably tumour-infiltrating lymphocytes play a crucial role in the antitumoural response of the host. Thus, these indices provided by these haematologic components may shed light on the host-tumour response [45–47].

Our results are consistent with the previous meta-analysis showing the prognostic value of NLR on resected gallbladder cancer by Saqib *et al* [49] and the prognostic role of NLR, PLR and MLR among gallbladder cancer patients in the meta-analysis conducted by Xu *et al* [38]. We obtained lower *I*^2^ values when Asian studies were analysed separately, which can partly explain the ethnicity and cut-off values as sources of heterogeneity. Worldwide, there is variation in the mortality rates for gallbladder cancer, with Asian countries such as Japan, Korea and Thailand among the top countries with high mortality rates [50, 51]. We included four studies conducted in the USA, which has 2–3 times lower mortality rates compared to other countries [52]. In addition to the differences in tumour biology based on ethnicity, there are differences in mean NLR among different countries [53, 54].

Differences in cut-off values also contributed to the heterogeneity among the pooled results. Thus, we utilised the median cut-off values in our subgroups and performed subgroup analyses on all biomarkers in contrast to the meta-analysis by Xu *et al* [38]. Using the median cut-offs for NLR and PLR decreased the heterogeneity. Subgroup analyses among studies with SII cut-off below 555 decreased the heterogeneity as well. Since the majority of the studies enrolled patients across stages I to IV, it was not possible to perform a subgroup analysis based on stage. This may also have affected the heterogeneity observed in the analysis of NLR, MLR and SII.

We here present the inherent limitations of our study. One of the major limitations and source of heterogeneity was the difference in cut-off values and different assays used in the determination of the peripheral blood counts. Due to the rarity of this disease, few studies were retrieved for inclusion in the study. In addition, it must be noted that most studies retrieved were derived from the Asian population limiting the generalisability of the results. The potential effect of differences between the populations studied such as age, sex and disease stage were also not investigated due to the majority of the studies investigating mixed populations. This meta-analysis primarily reviewed observational studies; hence, reporting bias which may have affected the results. The majority of the studies included did not enrol a control arm.

The results of the present study show the association of NLR, PLR and MLR with worse survival. These markers derived from the peripheral blood count are widely accessible, objective and with minimal cost. Moreover, these are promising prognosticators in gallbladder cancer, a disease characterised by poor prognosis, which may further guide treatment management. Since these tests have not been incorporated into routine practice, further prospective studies may validate their use in prognostication and treatment.

## Conclusion

NLR, PLR, MLR, PC and SII are promising haematologic biomarkers for worse survival in gallbladder cancer which can be used in prognostication and treatment guidance. Prospective studies are recommended to further evaluate their use.

## List of abbreviations

MLR, monocyte-to-lymphocyte ratio; NLR, neutrophil-to-lymphocyte ratio; PC, platelet count; PLR, platelet-to-lymphocyte ratio; SII, serum immune-inflammation index.

## Conflicts of interest

All authors have completed the ICMJE uniform disclosure form. The authors have no conflicts of interest to declare.

## Funding

No funding was received for this study.

## Ethical statement

The authors are accountable for all aspects of the work in ensuring that questions related to the accuracy or integrity of any part of the work are appropriately investigated and resolved.

## Author contributions

All the authors contributed equally to the research project and the final approved manuscript.

RNV: Conceptualisation, methodology, validation, original draft, writing – review and editing and final approval of manuscript.

HNT: Conceptualisation, methodology, validation, original draft, writing – review and editing and final approval of manuscript.

MSJ: Conceptualisation, methodology, validation, original draft, writing – review and editing and final approval of manuscript.

## Figures and Tables

**Supplementary Table 1.** Subgroup analysis according to median cut-off and region.

Subgroup analysis	#of studies	HR (95% CI)	*I* ^2^
Median cut-off			
NLR ≤ 2.675	8	1.965 (1.66, 2.32)	26%
NLR > 2.675	8	1.53 (1.23, 1.89)	57%
PLR ≤ 140.305	4	1.87 (1.52, 2.29)	0%
PLR > 140.305	5	1.37 (1.19, 1.58)	0%
MLR ≤ 0.29	3	1.96 (1.23, 3.11)	67%
MLR > 0.29	2	2.01 (1.18, 3.42)	62%
PC ≤ 252.5	3	1.24 (1.01, 1.51)	0%
PC > 252.5	2	1.13 (0.89, 1.45)	0%
SII ≤ 555	2	1.58 (1.33, 1.86)	22%
SII > 555	2	2.41 (1.12, 5.22)	72%
Region			
NLR (Asian)	13	1.77(1.51, 2.06)	47%
NLR (Non-Asian)	3	1.65(0.95, 2.89)	80%
PLR	Subgroup not possible since all studies are Asian		
MLR			
PC			
SII (Asian)	4	1.87 (1.41, 2.47)	51%
SII (Non-Asian)	1	1.37 (1.02, 1.84)	NA

**Table 1.** Characteristics of the included studies in the meta-analysis.

First author	Year	Study country	Study type	Sample size	Median follow-up	Outcome	Stage	NOS^a^	Cut-off value
Beal *et al* [23]	2016	USA	Retrospective	525, preoperative	NR^b^	OS RFS	None	4	NLR ≥ 5
Chen *et al* [18]	2021	China	Retrospective	93, preoperative	14 months	OS	I–III	4	SII ≥ 823.99 NLR ≥ 2.225 MLR ≥ 0.325
Choi *et al* [24]	2019	Korea	Retrospective	178, pretreatment	8.7 months	PFS, OS	III–IV	3	MLR > 0.24 NLR > 2 PLR > 108
Cui *et al* [25]	2018	China	Retrospective	159, preoperative	8.06 months	OS	I–IV	4	NLR ≥ 4.39 PLR ≥ 181.85 MLR ≥ 0.30
Deng *et al* [19]	2019	China	Retrospective	169, preoperative	21 months	OS	I–IV	3	NLR ≥ 2.61 PLR ≥ 145.33
Li *et al* [26]	2021	China	Retrospective	691, preperative	53.8	OS	I–III	6	SII > 510, NLR > 2.3, PLR > 144 PC > 300
Liu *et al* [27]	2021	China	Retrospective	303, preoperative	NR	OS	I–IV	3	PC > 205 NLR > 2.74 PLR > 136.84
Mady *et al* [28]	2020	USA	Retrospective	231, metastatic	NR	OS	IV	5	NLR ≥ 5
McNamara *et al* [30]	2014	USA	Retrospective	304, preoperative	14.4 months	OS	I–IV	4	NLR ≥ 3.0
Murthy *et al* [29]	2019	USA	Retrospective	419, preoperative, given preoperative chemotherapy	39.1	OS	I–III	3	SII > 900
Pang *et al* [31]	2015	China	Retrospective	316, preoperative	42 months	OS	I–IV	4	PLR ≥ 117.7 PC > 300
Sun *et al* [32]	2020	China	Retrospective	142, preoperative	NR	OS	I–IV	4	SII ≥ 600 NLR ≥ 2.50
Tao *et al* [33]	2018	China	Retrospective	84, preoperative	June 30, 2017	OS	III–IV	5	NLR ≥ 3.20 PLR ≥ 117.75 MLR ≥ 0.25
Wang *et al* [14]	2015	China	Retrospective	223, preoperative	NR	OS	I–IV	4	PC > 178
Wu *et al* [34]	2014	China	Retrospective	85, preoperative	16 months	OS	I–V	5	NLR > 2.3 PC > 200
You *et al* [20]	2019	Korea	Retrospective	173 patients, unresectable, given gemcitabine-cisplatin	8.6 months	OS, PFS, ORR	III–IV	4	NLR > 3 PLR ≥ 190
Zhang *et al* [17]	2019	China	Retrospective	419, pretreatment	NR	OS	III–IV	4	SII > 440
Zhang *et al* [35]	2016	China	Retrospective	316, preoperative	20.97 months	OS	I–IV	4	NLR > 2.61
Zhang et al [36]	2016	China	Retrospective	98, preoperative	NR	OS	I–IV	3	NLR > 1.94 PLR > 113.34
Zhu *et al* [37]	2019	China	Retrospective	255, preoperative	September 2017	OS	I–IV	4	NLR ≥ 3.13 PLR ≥ 143.77 MLR ≥ 0.29

a NOS Newcastle-Ottawa scale for cohort studies score

b NR not reported
